# Mutant SRF and YAP1 remodel the chromatin to entice cardiac myocyte nuclear division

**DOI:** 10.20517/jca.2022.25

**Published:** 2022-07-07

**Authors:** Ali J. Marian

**Affiliations:** Center for Cardiovascular Genetics, Institute of Molecular Medicine and Department of Medicine, University of Texas Health Sciences Center at Houston, Houston, TX 77030, USA

**Keywords:** Myocyte replication, transcription factor, hippo, regenerative medicine

Cardiac myocytes are responsible for the contraction and relaxation of the myocardium. Consequently, damage to the myocytes causes systolic and diastolic dysfunctions, which clinically manifest as heart failure with reduced or preserved ejection fraction, respectively. Despite being the quintessential cells in the heart, cardiac myocytes are susceptible to internal and external injuries resulting in death. Myocyte death is a relatively common phenotype in various myocardial pathologies, whether the cause is ischemia, a genetic disorder, viruses, or toxic agents. The loss is consequential and is compounded by the negligible replicative capacity of the post-natal cardiac myocytes, which are considered, by and large, terminally differentiated cells. The existing data suggest that ~1% of the cardiac myocytes are renewed every year, which is insufficient to offset the loss resulting from a significant myocardial injury, such as acute myocardial infarction^[1]^. In addition to lacking a clinically meaningful regenerative capacity, the myocardium also has a limited functional reserved capacity, as a loss of greater than 5% of cardiac myocytes is sufficient to promote left ventricular remodeling and dysfunction and a loss of > 25% is sufficient to cause cardiogenic shock and death.

Given the essentiality of adult cardiac myocytes to cardiac function and because of their negligible replicative capacity, a major focus of the investigators since the birth of modern molecular cardiology four decades ago has been to induce the mature cardiac myocytes to enter the cell cycle, divide and generate new myocytes. Numerous genetic and pharmacological interventions targeting the cell cycle regulators, transcription factors, and signaling pathways have been performed to entice the mature cardiac myocytes to replicate with partial success^[2–5]^. Despite the considerable insights gained through such interventions into the regulation of the cell cycle in the adult cardiac myocytes, the progress has been insufficient to advance the findings toward clinical applications to repair the injured myocardium and replace the lost cardiac myocytes.

In this issue of *The Journal of Cardiovascular Aging*, Xiao *et al.* report a *tour de force* approach to induce replication of cardiac myocyte nuclei^[6]^. The foundation of this impressive work stems from decades of investigations on the biological functions of serum response factor (SRF) and its role in cardiac development by Schwartz’s group and others^[7–9]^. The present study is sweeping in methodology and brilliant in approach. It utilizes state-of-the-art technologies to address the enigma of cell cycle arrest in adult cardiac myocytes. The study design is based on an in-depth understanding of the molecular biology of SRF and its role in cardiac development. The experiments reflect years of progress in understating cardiac myocyte differentiation and proliferation as well as the development of new techniques for transient gene expression, namely the synthetic modified mRNA constructs.

The foundation of this work could be divided into two pillars. The first pillar is the understanding of the role of SRF in the regulation of the expression of genes encoding sarcomere proteins. The second pillar is the knowledge of the role of YAP, the effector of the Hippo pathway, in cell proliferation. To elaborate, in the early 1990s, Schwartz and colleagues identified SRF as a transcriptional regulator of cardiogenesis. The group demonstrated that SRF, through its MADS box domain, binds to the CArG boxes in the promoter regions of genes encoding the sarcomere proteins and induces their expressions^[9]^. Induction of expression of the genes involved in cardiogenesis by SRF, however, requires recruitment of NKX2-5 and GATA4 transcription factors and their combinatorial binding to the CArG motifs^[7–9]^. Disrupting the combinatorial assembly of SRF, NKX2-5, and GATA4 on the CArD motifs substantially attenuates the transcriptional activity of the SRF in inducing the expression of the cardiac genes. These fundamental discoveries over the last four decades led the present investigators to hypothesize that blocking the binding of SRF to the CArG motif and inhibiting its interactions with NKX2-5 and GATA4 in adult cardiac myocytes will suppress the expression of the sarcomere genes and transit them into a premature state.

The second piece of the enabling discoveries pertains to the characterization of the Hippo pathway, a mechanosensitive contact regulated pathway, and its transcriptional effector YAP1 in regulating cell proliferation^[10]^. Activation of the Hippo pathway through a cascade of kinases phosphorylates YAP1, which leads to its cytoplasmic retention and suppression of transcription through the YAP1-TEAD pathway. In contrast, suppression of the upstream molecules of the Hippo pathway results in increased levels of the non-phosphorylated YAP, which translocates into the nucleus, binds to TEAD, and induces transcription of genes involved in cell proliferation^[11]^. Building upon this knowledge, a mutant form of YAP1, referred to as YAP5SA, has been constructed, which is resistant to phosphorylation by its upstream kinases and consequently localizes into the cell nucleus and induces expression of genes involved in the cell cycle progression^[11]^.

Tooled with the above knowledge, Xiao *et al.*, using the alanine scanning mutagenesis, generated a mutant form of SRF, referred to as SRF153(A3), also known as STEMIN, that neither recognizes the CArG boxes nor it interacts with NKX2-5 and GATA4 transcription factor^[6]^. The authors hypothesized that expression of the STEMIN will lead to suppression of expression of the sarcomere gene and, consequently, reprogram the adult cardiac myocytes to an immature state, which could be induced to proliferate upon the delivery of mutant YAP5SA [Figure 1].

Through a set of elegant studies, the authors demonstrate that STEMIN does not activate the cardiac-actin promoter and does not interact with NKX2-5 and GATA4 transcription factors. Having established the fidelity of the STEMIN, the authors then analyzed the genome-wide binding targets of STEMIN by ATAC-Seq and identified a distinct set of transcription factor binding sites, such as CTCF and IRF3 motifs but not the CArG sequences. Moreover, the authors complemented the ATAC-Seq findings with the transcriptomic data by showing that the expression of the mutant SRF failed to induce the expression of the myogenic genes. In contrast, STEMIN markedly induced expression of the stem cell markers such as *Nanog, Oct4*, *Dnmt1*, and *Dnmt2*, as well as cell cycle regulators *Cnna2, Cnnb1*, and *Cnne1*. Thus, the authors succeeded in generating and characterizing a mutant SRF protein that functions like a Yamanaka factor and reprograms the mature myocytes to an immature state.

Being aware of the potential fortuitous effects of the long-term expression of an active protein, the authors embarked on utilizing the recently established technology of synthetically modified mRNA (mmRNA) constructs to transiently express the STEMIN or SRF153(A3) in the neonatal rat cardiac myocytes (NRCM). The investigators successfully showed efficient and transient expression of STEMIN, which induced expression of the stem cell and cell cycle genes, consistent with their earlier data in the non-myocyte cells, whereas transcript levels of cardiac myocyte-specific genes were suppressed. Moreover, the authors provided evidence of DNA synthesis upon transient expression of STEMIN using the synthetic mmRNA in the NRCM, as indicated by the Edu assay showing DNA synthesis in 27% of the cells, consistent with the reentry of the NRCM into the S phase of the cell cycle.

Having achieved the objective of converting the NRCM to an immature nascent state, the investigators embarked on testing the next component of their hypothesis that the expression of the mutant YAP5SA in immature NRCM will entice them to proliferate. Accordingly, they transfected the NRCM with STEMIN as well as YAP5SA, using synthetic mmRNAs, and showed increased incorporation of Edu in the NRCM, indicating DNA synthesis. To advance these findings in the NRCM to adult cardiac myocytes, the main objective of the study, the investigators isolated adult mouse cardiac myocytes and transfected the isolated cells with the synthetic mmRNAs expressing the STEMIN and YAP5SA. Although both STEMIN and YAP5SA induced S phase DNA synthesis in the adult mouse cardiac myocytes, their co-expression led to the entry of these cells to the S phase of the cell cycle, as indicated by the Edu incorporation assay, in more than 90% of the cells. Moreover, the increased DNA synthesis was associated with an increased number of the diploid and tetraploid nuclei as well as an increased number of cells expressing DIAPH3 and showing cleavage furrows, which are markers for the anaphase and cytokinesis, respectively. Consistent with the increased cell cycle activity and cytokinesis, RNA-Seq data showed increased transcript levels of genes involved in spindle assembly and cytokinesis, whereas cardiac myocyte-specific genes were down-regulated. Furthermore, continuing in their elegant studies, the investigators analyzed chromatin accessibility upon transfection of the adult mouse cardiac myocytes with their synthetic mmRNA constructs by the ATAC-Seq and identified increased accessibility of the promoter regions of genes involved in the cell cycle, spindle assembly, and nuclear division in the transfected adult mouse cardiac myocytes. Closing the loop, the investigators analyzed the ATAC-Seq data and showed that the STEMIN has a distinct interactome compared to the wild-type SRF, including a strong preference for the CTCF, SP1, RBPJ, and TEAD1, among others. These findings further corroborated the initial discoveries in the non-cardiac myocyte cells.

The study has conceptual, scientific, and technical novelty. It is elegant in design and sophisticated in cardiac myocyte biology. The findings are remarkable and formidable for the evidence of cell cycle reentry, DNA synthesis, chromatin remodeling, activation of the replication fork, and replisome pathway, and conversely, suppression of expression of genes involved in sarcomere assembly upon expression of STEMIN and YAP5SA. The use of the synthetic mmRNAs to express STEMIN and YAP5SA is clever as it has a clear advantage in gene transfer and therapy studies because it not only provides an efficient expression of the intended protein(s) but also offers only a transient expression. The latter is desirable to avoid fortuitous effects resulting from a long-term expression of an exogenous active protein in target cells. Likewise, the mmRNA is not expected to translocate into the nucleus and cause untoward genomics effects.

The findings, as in any other discovery, also raise several intriguing questions that necessitate follow-up studies. Whereas the evidence of the cell cycle reentry of adult mouse cardiac myocyte upon transient expression of STEMIN and YAP5SA is robust, additional data would be required to demonstrate an increased number of the adult cardiac myocytes, i.e., myocyte proliferation. It is intriguing to speculate whether and how the cells that have entered the cell cycle upon the transient expression of these two transcription factors would exit the cell cycle to differentiate into mature myocytes and, if so, what would mediate their differentiation to maturity. Data on the effects of STEMIN and YAP5SA on cardiac myocyte contractile and relaxation functions as well as the metabolic state and electrophysiological maturity of the nascent myocytes would be valuable. Furthermore, whether the effects of STEMIN and YAP5SA in inducing nuclear division are additive or synergistic would require further documentation. One might speculate that these two transcription factors function sequentially, one inducing a primordial stage (STEMIN) and the other (YAP5SA) proliferation. Finally, it remains to be seen whether the findings of the *in vitro* studies also extend to the *in vivo* experiments and whether STEMIN and YAP5SA would prove to be beneficial in replacing the lost cardiac myocytes and repairing the damaged myocardium in larger animals and ultimately in humans. On the latter point, the investigators provide their initial set of *in vivo* studies in mice in an accompanying manuscript, which supports the potential utility of this approach in repairing the damaged myocardium^[12]^. The road to regenerative medicine is long and winding and yet studded with occasional impactful discoveries. The enthusiasts would be eagerly waiting for the follow-up studies, including studies in large models of myocardial injury, by Schwartz and colleagues and others.

## Figures and Tables

**Figure 1.**
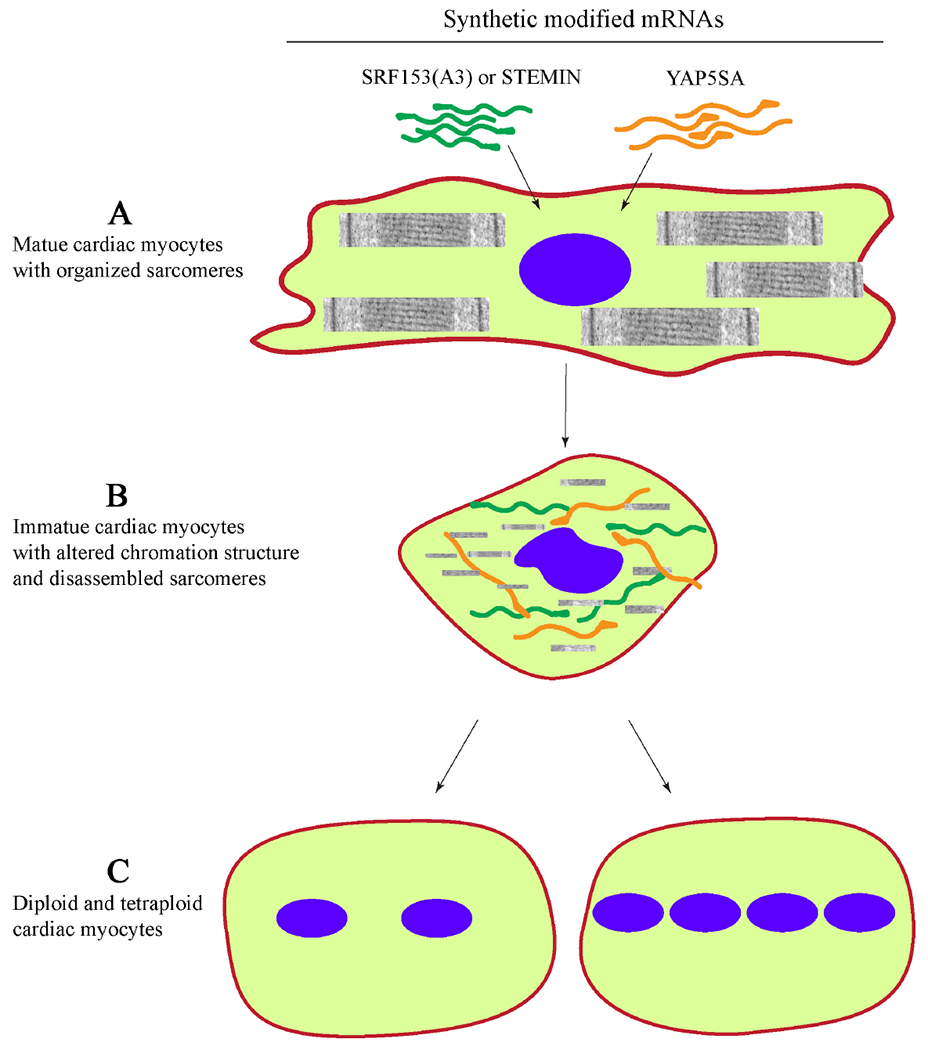
Replication of mature cardiac myocyte nuclei upon delivery of synthetic modified mRNAs expressing STEMIN and YAP5SA. (A) Transfection of mature cardiac myocytes with constructs expressing modified STEMIN [SRF153(A3) and YAP5SA]. (B) Disassembly of cardiac myocyte sarcomere structure, chromatin remodeling, and reversion to a immature state. (C) Nuclear division generating diploid and tetraploid myocytes.

## Data Availability

Not applicable.
